# What influences practitioners’ readiness to deliver psychological interventions by telephone? A qualitative study of behaviour change using the Theoretical Domains Framework

**DOI:** 10.1186/s12888-020-02761-3

**Published:** 2020-07-16

**Authors:** Cintia L. Faija, Janice Connell, Charlotte Welsh, Kerry Ardern, Elinor Hopkin, Judith Gellatly, Kelly Rushton, Claire Fraser, Annie Irvine, Christopher J. Armitage, Paul Wilson, Peter Bower, Karina Lovell, Penny Bee

**Affiliations:** 1 School of Health Sciences, Division of Nursing, Midwifery and Social Work, Manchester Academic Health Science Centre, University of Manchester, Manchester, UK; 2 Department of Psychology, University of Sheffield, Sheffield, UK; 3 Department of Language and Linguistic Science, University of York, York, Heslington UK; 4 Manchester Centre for Health Psychology, School of Health Sciences, University of Manchester, Manchester, UK; 5 Manchester Academic Health Science Centre, Manchester University NHS Foundation Trust, Manchester, UK; 6 Alliance Manchester Business School, University of Manchester, Manchester, UK; 7 NIHR School for Primary Care Research, Centre for Primary Care and Health Services Research, Manchester Academic Health Science Centre, University of Manchester, Manchester, UK

**Keywords:** Mental health, Telephone treatment, Guided self-help, Psychological treatment, Improving access to psychological services, Psychological wellbeing practitioners, Theoretical domains framework

## Abstract

**Background:**

Contemporary health policy is shifting towards remotely delivered care. A growing need to provide effective and accessible services, with maximal population reach has stimulated demand for flexible and efficient service models. The implementation of evidence-based practice has been slow, leaving many services ill equipped to respond to requests for non-face-to-face delivery. To address this translation gap, and provide empirically derived evidence to support large-scale practice change, our study aimed to explore practitioners’ perspectives of the factors that enhance the delivery of a NICE-recommended psychological intervention, i.e. guided self-help by telephone (GSH-T), in routine care. We used the Theoretical Domains Framework (TDF) to analyse our data, identify essential behaviour change processes and encourage the successful implementation of remote working in clinical practice.

**Method:**

Thirty-four psychological wellbeing practitioners (PWPs) from the UK NHS Improving Access to Psychological Therapies (IAPT) services were interviewed. Data were first analysed inductively, with codes cross-matched deductively to the TDF.

**Results:**

Analysis identified barriers to the delivery, engagement and implementation of GSH-T, within eight domains from the TDF: (i) Deficits in practitioner knowledge, (ii) Sub-optimal practitioner telephone skills, (iii) Practitioners’ lack of beliefs in telephone capabilities and self-confidence, (iv) Practitioners’ negative beliefs about consequences, (v) Negative emotions, (vi) Professional role expectations (vii) Negative social influences, and (viii) Challenges in the environmental context and resources. A degree of interdependence was observed between the TDF domains, such that improvements in one domain were often reported to confer secondary advantages in another.

**Conclusions:**

Multiple TDF domains emerge as relevant to improve delivery of GSH-T; and these domains are theoretically and practically interlinked. A multicomponent approach is recommended to facilitate the shift from in-person to telephone-based service delivery models, and prompt behaviour change at practitioner, patient and service levels. At a minimum, the development of practitioners’ telephone skills, an increase in clients’ awareness of telephone-based treatment, dilution of negative preconceptions about telephone treatment, and robust service level guidance and standards for implementation are required. This is the first study that provides clear direction on how to improve telephone delivery and optimise implementation, aligning with current mental health policy and service improvement.

## Background

Mental health problems affect approximately 110 million people worldwide; they are the main cause of disability and the third leading source of disease burden, after cardiovascular disease and cancer [1]. One in four people experiences mental health problems in a year; mental illness is the largest cause of disability in the UK and costs approximately £105 billion a year [2].

Depression and anxiety are the two most common mental health disorders [1]. Despite substantial advances in service provision, many people still find access to psychological therapies challenging [2]. To meet the increased demand of mental health, the NHS prioritises innovation to enable more people to receive cost-effective, evidence-based care and provide greater accessibility and choice [2].

Modern technology is changing how health care is delivered and has given rise to remote communication technologies including telephone, email, video-conferencing and Internet chat services. This is often labelled as telemedicine, telehealth, or tele-psychiatry [3–7], all of which have attracted considerable interest since the outbreak of the Global Covid-19 pandemic. Of these technologies, the telephone is arguably the simplest and most feasible, with a high likelihood of being implemented rapidly during times of crisis. It is also an accessible, National Institute for Health and Care Excellence (NICE)-recommended treatment option for mild to moderate depression and anxiety in routine practice [8, 9]. Systematic reviews demonstrate comparable effectiveness between [10–13], and adherence to [13], psychological treatments delivered by telephone and face-to-face, with robust analyses demonstrating the cost-effectiveness of telephone delivery [11, 14, 15].

Yet implementation in routine mental health services has been slow [15] and the adoption of telephone delivered healthcare remains a challenge [3, 16, 17]. CBT is considered particularly suitable for telephone administration because it is structured and skill based [18]; the therapeutic relationship is seen as important, but not sufficient, for optimal treatment outcomes [19]. Nonetheless concerns continue to be expressed about the use of telephone in mental health settings including, difficulties in developing a good therapeutic alliance, perceptions of reduced effectiveness, concerns about patient safety, lack of patient engagement and dropout [3, 20, 20–22]. Research undertaken in services, which are predominantly CBT based, identify particular concerns relating to the different ways of interacting over the phone (e.g. lack of non-verbal communication), that might lead to difficulties establishing an effective therapeutic relationship [21, 22]. However, a recent systematic review indicated a lack of support for this viewpoint; the telephone did not have a detrimental effect on the interactional aspects of psychological therapy when compared to face-to-face-delivery [23].

To date, no studies have sought to identify the key behavioural changes required to facilitate the implementation of telephone treatment into routine mental health care. The implementation of any new health technology is influenced by a number of factors [24], including and requiring explicit changes at individual, organisational and system levels [25]. Practical work is required when a shift towards new forms of normative conduct occur. At an individual level, deeply engrained beliefs around the intrinsic need for face-to-face interactions with patients and resistance to change may need to be challenged [3, 16, 26]. Additional organisational level barriers may include concerns around ‘cost’ and ‘reimbursement’ when adopting telemedicine [26].

We adopted the Theoretical Domains Framework (TDF), a conceptual base for understanding the determinants of behaviour change processes, to identify implementation problems and inform the design of interventions to improve telephone treatments [27, 28].

Using the TDF, we aimed to explore practitioners’ perceptions of the barriers and enablers to delivery of one NICE-recommended psychological intervention, i.e. guided-self-help by telephone (GSH-T) in the UK’s Improving Access to Psychological Therapies (IAPT) initiative. IAPT offers a stepped care model, in which GSH based on cognitive behavioural theory principles is delivered by Psychological Wellbeing Practitioners (PWPs) to clients with mild-to-moderate anxiety and depression. PWPs delivering low intensity ‘Step 2′ GSH provide treatment for the majority of referrals from primary care through its IAPT service [11] and thus have the greatest impact on service delivery. Whilst guidelines advocate telephone delivery at this level [8, 9] very little qualitative research explored the work required in implementing this practice change. Therefore, exploring the perspectives of practitioners delivering low intensity GSH interventions may highlight barriers to telephone delivery, despite evidence of clinical effectiveness,and offer further insight into the discrepancy between the treatment guidelines and routine clinical practice.

The aim of this theoretically informed qualitative study is to provide a comprehensive understanding of the challenges faced in clinical practice by PWPs to assist and enhance the implementation of telephone-based services.

## Method

### Study design

This was a qualitative study using semi-structured interviews, conducted prior to outbreak of the Global COVID-19 crisis. The epistemological position underpinning this study followed a social constructionist approach/paradigm, viewing knowledge as socially constructed rather than created [29–31].

An initial topic guide was developed based on prior literature by the research team and was reviewed by a Lived Experience Advisory Panel (LEAP). The LEAP comprised six people with lived experience of treatment for anxiety and/or depression, and/or family members engaged in the care of someone who had received psychological treatment. Some members of the group also had clinical accredited health professional training experience. The topic guide was informed by (but not restricted to) the Theoretical Domains Framework (TDF) [25]. The topic guide is presented in Additional File 1.

### Ethical approval

Ethical approval was granted by the North West - Greater Manchester West Research Ethics Committee (Ref: 18/NW/0372). Governance was approved at all participating IAPT (NHS and third sector) sites.

### Sample

Participants were eligible if they were trainees or qualified PWPs working in IAPT services, with or without experience of delivering GSH-T for anxiety and/or depression.

Sample size was defined following theoretical sufficiency criteria [32, 33], which was indicated by the thoroughness of data collection and data analysis providing detailed and differentiated categories/themes to a sufficient extent to inform domains of the Theoretical Domains Framework (TDF).

### Data Collection & Procedure

The study took place in North England, UK. Participants were recruited from three National Health Service (NHS) trusts and two third sector organisations that were commissioned to deliver IAPT services. One service (Service A) provided predominantly telephone therapy, with face-to-face-only offered where there was a specific need. At the other end of the spectrum, two services (B and C) offered predominantly face to face with telephone only offered when this was specifically requested by the patient or for the odd session. The remaining services (D, E, and F) offered a mix of telephone and face-to-face with some practitioners having half or full-day sessions (often working from home) that were dedicated to telephone work only. Services C and F were from the same NHS Trust but offered different service models.

Participants were invited to take part in an interview. Participants were recruited via a range of channels: direct invitation (email/letter), via their service manager, attendance by a researcher at team meetings, via advertisement on intranets, posters displayed at sites, or social media. Potential participants received a participant information sheet and a ‘consent to contact’ form by email or a paper copy. Participants interested in taking part returned the ‘consent to contact’ form to the research team, and researchers were available to respond to any questions. Upon agreement to take part, participants were asked to complete, sign and return an interview consent form via email or prior to participating in the study. At this stage participants also completed and returned a form including demographic details and experience in mental health service delivery. Data collection took place by telephone (participant preference over face-to-face).

At the beginning of the interview, the researcher reminded the participants about confidentiality, anonymity, and the voluntary nature of participation and the right to withdraw. Interviews lasted between 35 to 70 min. All interviews were audio-recorded and transcribed verbatim by an independent company. Any identifiable information was removed from the transcripts to protect participants’ anonymity. Data were securely stored at the Universities of Manchester and Sheffield.

### Data analysis

Data analysis commenced with inductive open coding [27] which was subsequently cross-matched to the Theoretical Domains Framework (TDF) [22]. Previous qualitative studies using the TDF used this data analysis strategy [34, 35].

Inductive analysis was performed by three researchers (CF^1^, CW, JC). The researchers read the transcripts to familiarise themselves with the data and then developed initial open codes for each of the transcripts. Fifteen percent of the transcripts (*n* = 5) were coded by two or three researchers (CF^1^, CW, JC) in the early stages of analysis to ensure consistency in the coding strategy. Agreement in codes and their definitions was reached through team discussion. Regular meetings were held between CF^1^, CW and JC to discuss these codes and emerging patterns.

The deductive analysis using the TDF consisted of four steps and involved five researchers (CF^1^, CW, JC, KA, EH). First, the researchers involved in this phase familiarised themselves with the TDF through regular consultation with experienced research team members, reading TDF guidelines and reviewing other literature where the framework had been utilised. Next, each of the codes identified through the inductive analysis was allocated to the appropriate constructs within the TDF domains, or positioned outside the framework if no relevant domain could be identified. Third, meaningful themes were developed within each TDF construct by grouping the allocated codes, and definitions of the themes were discussed and agreed by coders. As the TDF analysis progressed, allocated codes and developed themes were regularly presented to the wider research team and agreement on the TDF mapping was reached via team discussion. No TDF domains were excluded a priori but data have shown that some TDF domains are more relevant than others. Finally, an additional researcher from the study team (JG) checked 70% of the initial codes to ensure credibility of the dataset and analysis, checking codes against both the original verbatim quotes, and their TDF allocation.

Analysis was supported by QSR International’s NVivo-12 qualitative software [36].

### Quality

This study followed the consolidate criteria for reporting qualitative research (COREQ) [37] and accepted guidelines to ensure quality, validity and reliability in qualitative research [38, 39].

### Research Team & Reflexivity

Data were collected by CF^2^, JC, and KA. Data analysis was performed by CF^1^, JC, CW, KA, and EH. JG independently checked analysis for validity and reliability. Members of the wider research team provided reflective discussion, guidance and support through data collection and data analysis.

All researchers have experience in qualitative research. CF^1^ (PhD) is a Mental Health Research Associate with clinical experience of delivering therapy. JC (BSc) is a Research Associate in Mental Health Outcomes Research. CW (MSc) is a Service User Researcher with clinical experience of delivering therapy. KA (MSc) is a Research Assistant and a practicing PWP. EH (BA) is a Health Research Administrator. JG (PhD) is a Mental Health Research Fellow and Programme Manager. CF^2^ (MSc) is a Mental Health Research Associate.

No established relationship between researchers and participants existed prior to study commencement. At the interview, each researcher introduced herself, providing information about her current role and the reasons for doing the research. The research team reflected on potential bias, assumptions, and positions in relation to the research topic.

## Results

### Sample characteristics

From 37 practitioners expressing interest to take part, 34 provided consent and were interviewed. Of these, 28 described themselves as female. The majority (*n* = 30) self-reported as White British, and mean age was 32 years old (SD = 10, range 23–72). Further details about participants are presented in Table 1 including experience in telephone delivery and number of years working in mental health.

**Table 1:** Demographic details about practitioners (*N* = 34)

ID	Current Primary Role	Time in current role	Time working in mental health (years)	Telephone work experience	Service
P01	Trainee PWP	0–6 months	1–5	Moderate	A
P02	IAPT PWP	2–5 years	1–5	Moderate	B
P03	IAPT PWP	1–2 years	1–5	High	B
P04	IAPT PWP	1–2 years	1–5	High	B
P05	IAPT PWP	1–2 years	1–5	High	A
P06	IAPT PWP	6 months to 1 year	0–1	Moderate	A
P07	IAPT PWP	2–5 years	5–10	High	B
P08	IAPT PWP	5+ years	5–10	High	B
P09	IAPT PWP	1–2 years	1–5	Moderate	D
P10	Trainee PWP	6 months to 1 year	0–1	Moderate	D
P11	IAPT PWP	2–5 years	5–10	High	B
P12	IAPT PWP	0–6 months	5–10	Low	D
P13	Trainee PWP	6 months to 1 year	1–5	Low	D
P14	IAPT PWP	1–2 years	1–5	Low	D
P15	Trainee PWP	6 months to 1 year	5–10	Low	D
P16	IAPT PWP	5+ years	5–10	High	A
P17	IAPT PWP	1–2 years	1–5	Low	D
P18	IAPT PWP	1–2 years	1–5	Moderate	D
P19	IAPT PWP	2–5 years	1–5	Moderate-high	D
P20	IAPT Supervisor	0–6 months	5–10	High	C
P21	IAPT PWP	1–2 years	1–5	High	C
P22	IAPT PWP	5+ years	10–20	High	C
P23	IAPT PWP	2–5 years	1–5	High	C
P24	IAPT PWP	1–2 years	5–10	High	C
P25	IAPT PWP	0–6 months	1–5	High	E
P26	IAPT PWP	6 months to 1 year	1–5	High	E
P27	IAPT PWP	5+ years	10–20	High	E
P28	Trainee PWP	6 months to 1 year	1–5	Low	E
P29	Senior PWP Private Sector	0–6 months	1–5	High	E
P30	IAPT PWP	1–2 years	1–5	Moderate	E
P31	IAPT PWP	1–2 years	1–5	Low	E
P32	IAPT PWP	5+ years	10–20	Low	E
P33	IAPT PWP	2–5 years	1–5	High	E
P34	Trainee PWP	6 months to 1 year	1–5	Low	E

Our sample was comparable to demographic data reported on the 2015 IAPT census where the IAPT workforce was predominantly female (79%), White British (83%) and relatively young (66% < 46 years old) (2015 Adult IAPT Workforce Consensus report, NHS England & Health Education England).

### Theoretical domains framework

The initial open codes were categorised into 38 themes across eight domains of the TDF (see Table 2). The eight TDF domains that were endorsed were: knowledge, skills, beliefs about capabilities, beliefs about consequences, emotions, professional role, social influences, and environmental context and resources.

**Table 2:** TDF themes including barriers and enablers to improve delivery and aid implementation of guided-self-help delivered over the telephone (GSH-T)

TDF THEMES	BARRIERS	ENABLERS
**DEFICITS IN PRACTITIONERS’ KNOWLEDGE**	• Service centred drivers for the use of GSH-T	• Patient preference-driven approach to GSH-T (access, flexibility)
	• Lack of use of different modalities to deliver GSH	• Balance on the use of different modalities of delivery
		• Positive experience on telephone assessment facilitates telephone treatment
**SUB-OPTIMAL PRACTITIONER TELEPHONE SKILLS**	• Lack of telephone specific skills	• Developing verbal communication skills to deliver GSH-T through telephone specific training
	• Lack of quality assessment and monitoring on telephone delivery before and after becoming qualified	• Developing a warm and safe therapeutic environment
		• Moving to a positive attitude through practice, changes in negative beliefs and growth in self-confidence
**PRACTITIONERS’ LACK OF BELIEFS IN TELEPHONE CAPABILITIES & SELF-CONFIDENCE**	• Feeling less capable to develop a therapeutic relationship over the telephone compared to face-to-face	
	• Lack of self-confidence to work over the telephone related to the lack of visual and non-verbal cues	• Lack of visual increases sense of control over patient’s perceptions
**PRACTITIONERS’ NEGATIVE BELIEFS ABOUT CONSEQUENCES**	• Lack of effectiveness of telephone delivery regardless of the evidence	• Effectiveness of telephone delivery grounded on the evidence, practice and experience
	• Drop-out rates perceived to be higher for GSH-T (related to lack of patient engagement)	• Lack of visual helps to focus on patient’s verbal responses and increases efficiency
**NEGATIVE EMOTIONS**	• Feeling anxious and out of the comfort zone working over the telephone	
	• Feeling like a ‘*robot’* working over the telephone (lack of flexibility to deliver patient-centred care)	
	• Feeling overwhelmed, disconnected and burn out	
	• Feeling lonely and isolated	
**PROFESSIONAL ROLE EXPECTATIONS**	• Professional role varies pending on mode of delivery: coach vs therapist	
	• Delivering GSH-T perceived as a lower version of treatment	
	• Feelings of PWP role being undervalued	
	• Majority of telephone work done at Step 2 care only	
**NEGATIVE SOCIAL INFLUENCES**	• Negative preconceptions about telephone treatment	• Managing patient expectations
	• Patient expectations to receive f2f treatment	
	• Patient association of ‘*therapy’* with ‘*counselling’*	
	• Practitioner’s patient perceptions of telephone being ‘*not proper*’ therapy	
	• Lack of awareness of psychological treatments and its different modes of delivery	
**CHALLENGES IN THE ENVIRONMENTAL CONTEXT & RESOURCES**	• Working in a noisy ‘call centre’ with limited resources	• Informal peer support and supervision
	• Planning and preparation for telephone sessions is time consuming (before and after the session)	• Sessions over the telephone take less time (structure, focus, boundaries)
	• Lack of telephone-focused guidelines and service procedures for GSH-T	• Flexible working and/or improvements in working environmental conditions
	• Lack of formal supervision addressing challenges related to telephone delivery and telephone procedures	

#### Deficits in practitioners’ knowledge

Conflicts in practitioners’ knowledge of the origins, drivers, and processes of GSH-T emerged as an important influence on their engagement and readiness to promote or discourage remote treatment delivery.

Practitioners’ knowledge of the rationale for delivering GSH-T based on their university training, research evidence and NHS policy emphasised the value of using telephone delivery to improve access, reduce waiting times, provide flexibility, and offer patient choice. However, once in practice, practitioners perceived the move towards telephone treatments to be much more service-led, driven largely by a need to increase cost savings, improve service performance indicators, reduce waiting lists, and limit the need for physical space. Whilst the importance of each of these service-related issues was acknowledged, practitioners felt that services were imposing the telephone treatments ‘from the top down’, with little regard for patient choice or best practice. These perceptions limited practitioner enthusiasm for telephone use, and highlighted an explicit need for clear and transparent information related to its rationale:

*I suppose, the concern from my perspective is, are these decisions being made because they’re in the patient’s best interests, are these decisions being made because it’s going to be clinically more effective, or are they being made for other reasons, say, on a more financial basis, you know. Patients being seen quicker is important, but are we perhaps swinging a little too far to that. (P11, Male in his 20s, PWP, telephone experience high, Service A).*

Many practitioners were aware of a range of valid and credible reasons given by patients for preferring telephone delivery including work and child/family care commitments; physical conditions/disabilities; travel burden and anonymity. Practitioners suggested that offering a client the choice of telephone delivery, rather than imposing it as the only option, was more likely to be associated with good practitioner (but also patient) engagement and outcome.

Lack of patient choice was reported by some practitioners as a reason for moving away from the use of the telephone, towards a more balanced service that offered a variety of treatment modes. The majority of practitioners strongly valued working in services that offered multiple modes of delivery (e.g. group, face-to-face and telephone). In such contexts, positive experiences during telephone assessment provided a sufficiently secure knowledge base to encourage them to continue offering treatment remotely.

*So actually, having that assessment over the phone and actually seeing that it does go well and you manage to get through everything I think that then does open them up a little bit more to having telephone treatment. (P19, Female in her 20s, PWP, telephone experience moderate/high, Service B).*

#### Sub-optimal practitioner telephone skills

Data analysis revealed sub-optimal skills in telephone working and an urgent need to develop specific skills to enable a good therapeutic relationship, improve patient engagement, and effectively deliver GSH-T without visual aids and non-verbal cues.

Most practitioners reported a persistent lack of telephone-specific training, both within university courses and from in-service training opportunities post qualification. Training was often limited to one day or less and did not cover the different skills that practitioners felt they needed to deliver GSH-T. There was no difference in the level of telephone specific training between those participants who were currently trainees and those who had completed their formal training. Some trainees reported that the training they had received was wholly related to conducting assessments over the phone and not to interventions. Practitioners highlighted the value of formal training via lectures and workshops, but also suggested they would benefit from role playing, shadowing colleagues with experience, recording telephone sessions and telephone dedicated supervision.

*And being taught at university how to deliver assessment and interventions, we weren’t taught how to do it on the telephone. So that was just a general worry that I hadn’t been taught that way and it was going to … I wasn’t going to be able to do it. (P29, Female in her 20s, Senior PWP from private sector, telephone experience high, Service F).*

Practitioners highlighted that telephone specific skills training should aim to develop non-verbal communication skills, including effective use of tone of voice, sounds, pauses, and silences to convey empathy; but also improve verbal skills to encourage a natural and flowing conversation and equip practitioners with the necessary skills to manage more or less talkative patients assertively. These skills were considered beneficial in establishing an effective therapeutic bond, sufficient to facilitate client engagement and ensuring the delivery of a high quality service.

Practitioners’ communal discourse emphasised a need to be assessed and monitored in the delivery of GSH-T throughout training and clinical practice, before and after becoming qualified. Practitioners with more experience of telephone treatment reflected on the development of their telephone skills over time and their consequent changes in attitude and growth in self-confidence.

Those with more telephone experience explained that they build up a therapeutic environment and alliance via active listening, being verbally empathetic by using the patient’s name more often than they would face-to-face, referring to information from previous sessions, and avoiding the use of jargon. To compensate for the lack of visual cues, practitioners reported asking more questions, explaining materials and homework in more detail, using reflections and metaphors, and adapting language to aid patient understanding.

*I think with it being over the phone you have to talk through the materials in a lot more detail […*] *just be more descriptive and using metaphors maybe (P20, Male in his 40s, IAPT Supervisor, telephone experience high, Service E).*

#### Practitioners’ lack of beliefs in telephone capabilities and self-confidence

Practitioners reported beliefs about being less capable and confident to effectively deliver treatment over the telephone compared to face-to-face, with these beliefs engendering feelings of anxiety, and interfering with the quality of treatment delivery and practitioners' enthusiasm for working by telephone. Some practitioners reported lack of confidence delivering telephone treatment but not telephone assessments. A minority of trainees highlighted the potentially enabling influence of previous experience on these attitudes, suggesting that less familiarity with face to face work and/or prior experience of telephone working (e.g. through Samaritans or market research roles) made telephone delivery less daunting. The majority of practitioners perceived themselves as being less capable of working collaboratively with patients over the telephone than face-to-face due to feeling that sessions were more scripted and impersonal over the telephone. Practitioners also reported more difficulties building a therapeutic alliance over the telephone compared to face-to face, and related this to communication difficulties that challenged their ability to convey empathy and compassion.

*R: … I feel really confident with telephone assessments but the treatment is something that I find more difficult over the phone. […*] *I think perhaps with an assessment it’s not sort of guaranteed that you’re taking that person on for treatment and it is very much about information gathering, you know, and getting information that you need to be able to help get that person to the right place. Whereas when it comes to treatment I think it’s really important to be able to build that rapport with that person and that good therapeutic relationship which I think is quite hard over the phone. (P26, Female in her 20s, PWP, telephone experience high, Service F).*

Practitioners typically felt less capable of delivering GSH-T because of the lack of visual aids and non-verbal cues (e.g. facial expressions, eye contact). This interfered with practitioners' own professional values of a ‘good therapist’ and delivering ‘good quality’ therapy. Many practitioners reported a lack of confidence and skills to facilitate patient understanding while working through different elements of the treatment without being able to point to a diagram.

*So not visually seeing the patient, not being able to … I don’t know, see their body language, things like that, their expressions. I think just general worries like that, about delivering interventions […*] *because the university didn’t prepare us for that. (P29, Female in her 20s, Senior PWP from private sector, telephone experience high, Service F).*

On the other hand, two practitioners perceived that the lack of visual cues increased patients’ perceptions of practitioner competence, which ultimately resulted in feeling less pressured and more self-confident.

*But I also find that it’s easier for me because, I suppose because maybe the clients not watching me and the client’s aren’t seeing what I’m doing. So I can have all of my information in front of me, I can read from things if I need to. […*] *In my face-to-face sessions I feel like there is an element of having to seem like I know what I’m talking about and to be able to just talk about it. Whereas in a telephone session I can get that across, I can portray that confidence without sensing that I’m reading from a sheet or I’m reading from some notes I’ve made and I can be surrounded by information when I’m on the phone. Whereas I can’t do that in a face-to-face session. (P05, Female in her 20s, PWP, telephone experience high, Service D).*

The majority of practitioners reported how their initial negative attitudes to telephone work shifted to a more positive approach through practice. Experience and time allowed practitioners to discover that their initial negative views of GSH-T were not always a reflection of reality. Discovering that GSH-T worked effectively for patients and receiving positive patient feedback, promoted engagement and increased self-confidence.

*Just doing it, basically. The more you do, the more comfortable, you know, the more I’ve done, the more I’ve thought, yeah, this doesn’t seem to be a disadvantage for the patient. (P16, Female in her 70s, PWP, telephone experience high, Service D).*

#### Practitioners’ personal negative beliefs about consequences

Beliefs on lack of capabilities and self-confidence to deliver GSH-T play an important role in the maintenance of practitioners’ beliefs about telephone treatment being less effective compared to face-to-face. Data highlighted that holding positive beliefs about effectiveness of telephone treatment increases engagement towards this mode of delivery.

Practitioners' perceptions related to the lack of effectiveness of GSH-T compared to face-to-face were attributed to the lack of beliefs on capabilities and self-confidence to deliver an effective intervention without using visual aids.

*The visual stuff really helps* [to deliver psycho-education]*. So, I think doing that over the phone might be quite difficult, and obviously that might affect how effective the treatment is as well. (P34, Female in her 20s, Trainee PWP, telephone experience low, Service F).*

On the contrary, two practitioners perceived the lack of visual and non-verbal communication as facilitative to delivering GSH-T allowing more focus on patient verbal responses, preventing misinterpretations, and thus increasing intervention quality and efficiency.

The vast majority of practitioners believed dropout rates were higher in GSH-T compared to face-to-face delivery and that recovery rates were lower, predominantly due to the lack of patient engagement with this mode of delivery. However, only a few practitioners identified factors associated specifically to the telephone mode to explain lack of engagement. These included lack of visual cues, poorer quality of therapeutic alliance, anonymity, patient expectations of receiving face-to-face treatment (instead of telephone) and counselling (rather than GSH), telephone appointments considered less important and easy to cancel, lack of a warming environment and reduced opportunity to talk openly about different difficulties. Some practitioners emphasised that telephone treatment may be more difficult for patients due to the reliance on patients’ organisational skills (e.g. having session material to hand), which may also negatively influence engagement.

*I think in previous experiences that some person with telephone treatment sessions, the people can drop out a little bit earlier. I’m not sure that if that would relate to, like I said, a therapeutic alliance; not being able to see the person, but finding it maybe a little bit more difficult. (P09, Male in his 20s, PWP, telephone experience moderate, Service B).*

*I think it’s probably easier for patients to drop out or to disengage or DNA when it’s a telephone appointment. Maybe it’s not something as worthwhile or as important as significant as going to attend at a surgery or going to attend face-to-face. (P29, Female in her 20s, Senior PWP from private sector, telephone experience high, Service F).*

However, there were also commonalities between telephone and face-to-face to explain lack of patient engagement, including: low treatment motivation, timing not being ‘*right’* for treatment, patient and/or practitioner perceptions of lack of patient suitability for GSH treatment, and practitioner characteristics not meeting patient expectations.

Perhaps predictably, practitioners who held beliefs that GSH-T had the potential to be equally as effective as face-to-face (based on evidence and clinical practice) and perceived the telephone as a way to aid attendance and reduce cancellations rates were more engaged with this mode of delivery.

#### Negative emotions

Deficits in telephone skills, perceived lack of capabilities and lack of assessment and monitoring, and perceptions of reduced patient-centeredness and service effectiveness all combined to negatively influence practitioners’ emotions towards GSH-T. Practitioners reported feeling anxious, overwhelmed, burnt out, disconnected, lonely and isolated working over the telephone. Over time, these feelings appeared to diminish as practitioners became familiar with telephone treatments.

A few practitioners reported that working over the phone felt ‘*cold’* and emotionally detached and were therefore cognisant of the risk that sessions may become impersonal or appear scripted. When discussing telephone work, some practitioners described feeling ‘*robotic’* and lacking spontaneity and authenticity, influencing a less collaborative approach.

*Sometimes as well when you’re doing things over the phone, especially when you’re trying to describe things, it feels more directed. Whereas if you can just kind of show someone an image of something or a diagram and just say, you know, what are you getting from this, it kind of feels more collaborative. (P25, Female in her 20s, PWP, telephone experience high, Service F).*

A few practitioners, who described their work as isolated and lonely, suggested that those feelings became more entrenched once the majority of the day involved working over the telephone and they became home-based.

#### Professional role expectations

Professional role expectations were related to delivering treatment using the traditional face-to-face mode instead of working with a telephone headset. However, the vast majority of practitioners reported they delivered telephone-treatment if they needed to do so; but perceived this mode of treatment was not equally valued as face-to-face.

The majority of practitioners held the belief that telephone delivery is viewed by others as appropriate for low intensity interventions (i.e. GSH) delivered by low intensity practitioners (PWPs) but not for those delivering higher intensity interventions (e.g. cognitive behavioural therapy).

*I think especially higher intensity therapists as well, very rarely have I met anyone that does any telephone that way really, on a regular basis. (P33, Female in her 20s, PWP, telephone experience high, Service F).*

Practitioners questioned why services did not feel it was appropriate for all mental health practitioners to use the telephone on a regular basis if the main aim for its use was to improve access to psychological treatment. A common perception in practitioners’ narratives was that the use of the telephone was instead suggestive of an unwelcome staff hierarchy within IAPT, and a potential indicator of professional credibility. This may not only influence perceptions of telephone treatment as being inferior but also practitioner feelings of being undervalued.

*Some people do see it as, this is a resource issue and it just seems really, I don’t know, really foreign and sometimes, I don’t know, second best. (P04, Female in her 40s, PWP, telephone experience high, Service A).*

Even though practitioners described the treatment delivered over the telephone as the same as face-to-face, practitioners role expectations were challenged when their working responsibilities changed from delivering psychological treatment in a private office face-to-face to working remotely at a desk with a telephone headset. Although the majority of practitioners reported that they ‘*did not mind*’ delivering GSH-T if they needed to do so along with other modalities, this role was perceived much more as educational coach/mentor than an experienced psychological practitioner.

*And I don’t know whether it’s a perception that, you know, because it’s telephone, is it just a little bit of a chitchat, is it proper therapy, so to speak, I don’t know. (P08, Female in her 30s, PWP, telephone experience high, Service A).*

#### Negative social influences

Practitioners reported negative social influences towards telephone treatment and GSH. Data suggests that clarifying patient expectations early on in treatment may enable patient engagement.

Practitioners believed that patient preferences for psychological treatment were influenced by a pre-conceived notion of ‘*therapy’* as ‘*counselling’* and a socially embedded expectation that this would be achieved face to face. These expectations were believed to be reinforced by GPs and depictions of talking therapies in the media. GSH-T, as a remotely delivered intervention directly contradicted this vision of treatment.

*You know, we get the same quite negative response from quite a lot of people and I think it’s just a perception of you have a one-to-one with one therapist and it’s almost maybe like a media image of what counselling or therapy is. (P03, Female in her 30s, PWP, telephone experience high, Service A).*

Practitioners’ perceptions of patient preferences for face-to-face treatment appeared to be interlinked with their own expectations and preferences for service delivery. Practitioners expressed their concerns about how telephone treatment may not be taken seriously by patients and would not be perceived as ‘*proper’* therapy.

*I couldn’t see the patient and they would feel that they weren’t getting a proper treatment […*] *they’re not taking it as seriously because it’s on the telephone. (P29, Female in her 20s, Senior PWP from private sector, telephone experience high, Service F).*

#### Influences of environment and need for resources

Delivering telephone treatment in a noisy environment with limited resources and a lack of standardised guidelines to work remotely increased anxiety, compromised credibility/value, and interfered with practitioners’ engagement and delivery.

For some practitioners, working in a shared open plan office to deliver telephone treatment was described as a noisy ‘*call centre’*, and did not match professional role expectations of working in a clinical setting that ensured privacy and confidentiality.

*I mean, some big services, like my last service, you do feel like you’re in a bit of a call centre environment, not here the service is really well managed here. But, nevertheless, you do think, I’ve been sat at a desk with a headset on for the last eight or nine hours, it’s not perhaps what you came into the job to do. (P11, Male in his 20s, Trainee PWP, telephone experience high, Service A).*

On the contrary, other practitioners valued the fact that working in an open plan office facilitated immediate support and advice from colleagues (informal supervision), improving quality of telephone delivery.

Working from home was an option favoured by many practitioners and regarded as an incentive for delivering telephone treatment. It not only provided a quiet, private and confidential space but also had other personal benefits (i.e. better work-life balance). The disadvantage of home working included feelings of isolation and loneliness, and perceptions of managing patient risk as being more challenging.

*so that’s why I choose to do it from home. It’s really quiet […*] *it really works and it gives me time to … do that listening (P27, Female in her 40s, PWP, telephone experience high, Service F).*

*I think the only thing that is an issue, but it’s been put across by many PWPs, lone working is...you can go weeks without seeing any colleagues. Because, if you’re not in a clinic with....if you’re working from home you don’t bump into people. (P23, Male in his 30s, PWP, telephone experience high, Service E).*

The policy for working from home varied across services and working in a private office was usually not possible due to limitations on physical space. Rooms with soundproof board/cubicles were suggested to reduce noise and facilitate privacy. In addition, practitioners emphasised the importance of good quality headsets, mobile phones or telephone landlines because limited access to adequate resources increased stress.

*We didn’t have enough telephones or enough headsets or enough chairs and especially with agile working it was kind of a case of first come first served, so that was sometimes difficult […*] *we had mobile phones and headsets with those but you couldn’t really hear and the patient couldn’t hear you so that didn’t really work […*] *I think it would be good to actually have your own little cubicle or whatever so you could do it privately; obviously that’s not always feasible […*] *(P26, Female in her 20s, PWP, telephone experience high, Service F).*

A lack of telephone-focused guidelines and service procedures addressing the differences encountered when delivering telephone treatment emerged from the data and influenced practitioners’ engagement and delivery. Practitioners reported lack of clarity on how to share materials with patients (e.g. how/when/what format), how to administer outcome measures in a clinically meaningful way, how to manage homework and patients not having session materials to hand (e.g. proceed with the session or reschedule). Further protocols were needed to outline how to manage the patient environment (i.e. a public/noisy location), respond to patients undertaking other activities during calls (e.g. driving, doing the washing up), manage risk if contact is cut (e.g. patient hangs up).

*But I think I would hope that it would come with having been thoroughly thought through with, you know, things like … that’s all very well and good but how do we get information out to people? […*] *we’ve now got to find the time and remember to post that information out or to email it out, or get them to collect it, whatever it might be. (P32, Female in her 30s, PWP, telephone experience low, Service F).*

We found inconsistencies in practitioners’ procedures depending on the modality of delivery. For instance, when managing homework non-compliance a few practitioners felt entitled to re-schedule the session if this occurred over the telephone whereas face-to-face they would proceed.

Practitioners reported an increase in preparation time for sessions delivered by telephone in comparison to face-to-face. This was related to the need to plan sessions well in advance and to posting materials to patients, which influenced their enthusiasm to work remotely. The use of email (instead of post) to share materials with patients and the use of a workbook including all the potential information needed for treatment was favoured to reduce the time allocated to planning and posting. However, using a workbook could be perceived as not being patient-centred.

On the other hand, the majority of practitioners reported that sessions conducted over the telephone were usually quicker or delivered within the estimated time, and the administration time was reduced because notes were completed during the session. We found telephone delivery was more conducive to implementation of boundaries, maintenance of focus and adherence to protocol, which was noted as helpful due to time constraints.

*I suppose from a time management point of view it’s easier to stay on track time wise and not let the conversation drift as it does a bit face to face. (P07, Female in her 30s, PWP, telephone experience high, Service A).*

## Discussion

Although health policy is shifting substantially towards the introduction of remotely administered care, research has identified several challenges to the implementation of psychological interventions by telephone [3, 40]. This study explored the factors that interfere with or promote delivery of GSH-T in mental health services from practitioners’ perspectives. This analysis was facilitated by the TDF, which utilises psychological theory to understand behaviour change processes and helps to translate changes effectively into routine and responsive care. Our research aligns directly with mental health policy mandating to increase patient access, choice, quality and integration and findings provide evidence of how to bridge the gap between research and practice in telephone delivery.

Previous qualitative research on telephone delivery highlighted significant concerns around therapeutic alliance; effectiveness and drop out; and patients' risk, expectations and engagement [3, 14, 20–22, 41–43]. Our findings are in line with previous research but this is the first study that provides specific recommendations on how to improve telephone delivery and contribute to integrating research and clinical practice (implementation).

Eight of the 14 TDF domains emerge as relevant, i.e. knowledge, skills, beliefs about capabilities, beliefs about consequences, emotions, professional role, social influences, and environmental context and resources; suggesting a multicomponent intervention to improve delivery and implementation of GSH-T. TDF domains were interdependent to some degree highlighting that improvements in one domain could lead to secondary advantages in another. For example, an improvement in skills would likely lead to increased belief in capabilities and confidence which would in turn impact on practitioners' belief about the consequences and any negative attitude towards telephone therapy. The cumulative effect of these changes has the potential to improve the patient’s experience of telephone therapy and lead to more positive outcomes. Participant responses were not quantified across our sample as this was not appropriate for the qualitative methodology we adopted. However, the high value that practitioners attributed to therapeutic alliance was a recurrent observation. This was evident in themes relating to practitioners’ values and professional identity, their perceived capability to conduct telephone work; their beliefs about the consequences of telephone delivery; and the importance of optimising training and skills adaption (e.g. conveying empathy) to support telephone delivery. The salience of this topic across multiple TDF domains highlights a potentially valuable focus for a multi-component intervention.

Based on our findings, changes should focus on patient, practitioner, service, and community levels. Our research study provides evidence of how to extend the reach and effectiveness of telephone treatment focusing on five target areas of change:

1. align theoretical knowledge drivers of GSH-T into clinical practice
2. develop practitioners' telephone skills
3. challenge negative preconceptions about telephone treatment
4. adjust professional working environment and increase resources
5. adapt guidelines and standardise procedures for telephone delivery

### Target 1: Align theoretical knowledge drivers of GSH-T into clinical practice.

Mental health policy [44] and NHS England’s Five Year Forward View for Mental Health (2016) [2] propose that mental health services develop new ways of working focusing on access, quality and integration. However, our findings have shown that practitioners’ perceptions about the use of GSH-T in clinical practice was service-led and driven by the need to increase cost savings, reduce treatment waiting times, pressures to meet service performance targets and lack of physical space. A service-driven approach instead of a patient-driven one to deliver telephone treatment limited practitioner and patient enthusiasm to engage with remote delivery. Thus, services need to ensure that telephone treatment is aligned with patient choice and is not the only option of care. If telephone is the only mode of delivery, a clear rationale for its use and a transparent approach are needed to set real expectations and improve engagement. These findings are in line with recently published research conducted with decision-makers in mental health, which highlights the importance of providing clinical motives for the use of remote delivery in IAPT services to promote practitioners’ engagement [45].

### Target 2: Develop practitioner's telephone skills.

Findings from our study highlighted deficits in telephone skills. There is a need to develop skills to deliver telephone treatment, including: *how* to deliver a patient-centred treatment when following a manualised approach, *how* to assess and manage patient risk, *how* to deliver an effective treatment without the use of visual aids, *how* to use silences, *how* to build up a safe therapeutic environment, *how* to convey empathy and develop a good relationship, and *how* to increase patient engagement within and between sessions. In addition, assessment of telephone-skills during professional training and following qualification were identified as important, as well as ongoing telephone specific supervision addressing telephone-delivery challenges and telephone-performance.

Therefore, recommendations should be specific to trainees and qualified PWPs to ensure the development and application of telephone specific skills is accessible throughout the course of their professional role. The development, assessment and monitoring of practitioner’s telephone skills could be addressed providing telephone training before (IAPT University courses) and/or after qualification (IAPT services). Furthermore, services need to ensure opportunities for continued professional development of telephone skills.

Our findings indicate that the development of telephone skills has the potential to improve practitioners’ self-confidence, decrease levels of anxiety, and thereby facilitate quality of delivery and implementation of telephone treatment. Emphasis on telephone skills training could influence practitioners’ views on the credibility and importance of telephone delivery, and facilitate engagement.

### Target 3: Challenge negative preconceptions about telephone treatment.

Findings from our study highlight negative preconceptions about telephone treatment from practitioners, patients and community. These were mainly related to the socio-cultural idea of therapy being associated to ‘counselling’ and face-to-face delivery, revealing the lack of knowledge on different types of talking therapies and modes of delivery. The conflict between expectations and the psychological treatment offered/received highlights the need for increasing awareness at a patient and community levels. Regarding GSH-T in particular, information should emphasise that qualified practitioners deliver this treatment, and that is equally effective as face-to-face. In addition, clarification of patients’ expectations and treatment procedures (attendance, cancellation, discharge, confidentiality, environment, and boundaries) should be addressed at the initial assessment and/or early on in treatment to prevent misunderstandings/disappointment as well as to facilitate engagement with telephone treatment. These findings are in line recent findings that identified that patients referred to mental health services are often unaware about remote delivery and have unrealistic expectations of what their treatment might involve [46].

Our findings highlighted that despite professionals’ knowledge on the effectiveness of this mode of delivery they hold beliefs about lack of effectiveness, the difficulty of developing therapeutic alliance, lack of perceived capabilities to work by telephone, and that telephone is an inferior version of treatment. This suggests the need to move from providing evidence on telephone treatment to challenge practitioners’ beliefs by improving telephone skills and by promoting personal reflection and discovery through training, practice and supervision. The development of a positive attitude (through means aforementioned) may increase engagement in, and the quality of, telephone delivery; this in itself establishing and reinforcing the practitioners’ newfound positive beliefs.

### Target 4: Adjust professional working environment and increase resources.

Practitioners working in a noisy ‘*call centre’* setting with lack of resources described increased levels of anxiety and concerns about confidentiality and privacy. This type of environment and the high volumes of telephone work conflicted professional role expectations and subsequently deterred the practitioners’ enthusiasm. Findings suggest that working in a comfortable environment that supports remote working is extremely important to improve engagement and quality of delivery. The clinical setting should promote peer support to learn from each other and ensure collaborative working. Where possible, services should consider providing a balanced approach towards different modes of delivery (e.g. face-to-face, telephone, group), flexible working and choices (e.g. home-based, small or private offices), and good quality of resources (e.g. headsets, laptops, landlines). In addition, an increase in time allocation for planning and preparation for telephone delivery should be acknowledged.

### Target 5: Adapt guidelines and standardise procedures for telephone delivery.

Findings suggest implementation should account for the adaptation of guidelines and standardise procedures to deliver telephone treatment. These procedures should provide clear guidelines in relation to: managing patient risk remotely, managing communication, correspondence and engagament when patients are in a potentially non-confidential environment, losing telephone connection, managing patients non-response to phone calls and between session work, and sharing psychoeducational materials with patients. Clarification on procedures to deliver telephone treatment would help to not only facilitate and standardise implementation of GSH-T, but also to reduce practitioners’ anxiety, improve confidence, promote change in preconceptions and enhance credibility towards telephone working.

It is important to highlight that in addition to the five identified areas of change, several factors were acknowledged as facilitators of delivery of GSH-T. These factors included: increased efficiency of telephone treatment due to maintenance of focus and structure within sessions; easier implementation of boundaries (compared to face-to-face); practitioner anonymity as a way to prevent misjudgements from patients and feel more confident; practice as a facilitator of positive experiences, and, for some practitioners, the lack of visual and non-verbal language supported attention to patient verbal responses. Some of these findings are comparable to the advantages practitioners highlighted when delivering assessments (not treatment) over the telephone [43].

Interestingly, six of the 14 TDF domains appeared to be less relevant to improving telephone delivery, i.e. memory/attention/decision processes, behavioural regulation, optimism, intentions, goals and reinforcement. The absence or limited data within these domains lead us to hypothesise that the two domains related to cognitive processes (i.e. memory/attention/decision processes and behavioural regulation) are not dominant barriers interfering in the delivery or implementation of telephone treatment; however, changes in the working environment would positively influence attention, concentration and facilitate active listening. The lack of optimism, intention, goals and reinforcements to use telephone-treatment are hypothesised to be influenced by deficits currently addressed within the five areas identified for change. Thus, an intervention targeting the suggested areas of change could prove beneficial in increasing optimism and improve practitioners’ motivation to work remotely.

Evidence of promoting professional behaviour change in healthcare has found that the best chance of success includes an intervention targeting individual, community and population levels simultaneously and consistently [47–50]. Therefore, a multifaceted intervention that addresses all five highlighted areas of change may be most conducive to improving telephone delivery, efficiently maximising the likelihood of effective implementation in routine care. At a minimum, developing practitioner telephone skills to deliver support remotely, challenging negative preconceptions of telephone as a lower version of therapy, increasing awareness of telephone treatment, and the provision of robust guidance and standards should be prioritised for implementation. These areas were found to influence PWPs’ levels of anxiety, self-confidence and perceived capability to work via the telephoneand address their beliefs regarding the effectiveness of GSH-T and the perceived flexibility of the PWP role and the modes of delivery it can encompass.

### Limitations and strengths

There are limitations to this study that warrant discussion. Although face-to-face interviewing was offered for data collection, all participants elected to be interviewed over the telephone. This may mean that our study participants were naturally more comfortable with and experienced in talking over the telephone. All participants had some experience of telephone work, which is important to note when considering implementation challenges. Only a small number of participants (*n* = 6) described a “low” level of experience in delivering GSH-T. Most participants were able to recall their feelings and experiences from when they first started to use the telephone to deliver interventions. Although the interviews were conducted via telephone and the interview transcripts were anonymised, it is important to consider potential participant bias towards socially desirable responses. Data related to patient attitudes were practitioners’ perceptions and may not reflect patient views.

There are several strengths of the study. The sample is comparable to the IAPT workforce. In addition, practitioners from different services with diverse levels of experience delivering GSH-T were interviewed, providing a wide range of views. Although the interviews focused on the PWPs' experience of telephone delivery within their role, information was also provided in terms of contrast between face-to face and telephone delivery as well as information regarding online interventions. PWPs also discussed themes related to broader aspects of delivering psychological interventions, such as, person-centred care and maintaining client engagement which can arguably inform not only other telephone delivered interventions but also other modalities such as computerised guided self-help. The involvement of multiple researchers in data analysis from a range of backgrounds reduced individual bias in interpretation and will have enhanced the quality of our analysis.

This research was conducted prior to the 2020 global COVID-19 outbreak, but has particular relevance to the health service challenges that have been imposed by it. Responsive national measures, including social isolation and distancing have resulted in an increased and urgent need to implement remotely delivered interventions into routine practice. Our findings highlight the importance of fully addressing practitioner concerns regarding the comparable safety and effectiveness of remote and face-to-face services, and the potential influence of professional role identity and perceived intervention ‘fit’. The inevitable increase in the delivery of psychological interventions by telephone during this time may provide the opportunity for a range of mental health professionals to gain experience of this mode of delivery which may help to challenge preconceptions and shift some of the misconceptions regarding this way of working, and patients preferences and acceptance of alternative models of service access. .

## Conclusion

There is existing evidence base presenting both the quantitative evidence of psychological interventions delivered by telephone being clinically effective for mild-moderate anxiety and depression, and a smaller qualitative evidence base often presenting data from selective samples or expert opinion challenging the process. This is the first study to address the gap between the evidence and the use of telephone treatment providing a clear direction on how to improve telephone delivery and optimise implementation, which has relevance for growing population demand, policy initiatives, and health technology integration. Furthermore, our work has attracted considerable importance since the outbreak of the Global Covid-19 pandemic and could prove fruitful to face the current needs that demand moving from face-to-face to remote delivery models.

## Supplementary information

**Additional file 1.** Topic Guide.

## Data Availability

The dataset generated and analysed during the current study are not publicly available due to potential for breach of anonymity, but are available from the corresponding author on reasonable request.

## Abbreviations

GSH: Guided-self-help
GSH-T: Guided-self-help delivered by telephone
IAPT: Increasing access to psychological therapies
LEAP: Lived Experience Advisory Panel
TDF: Theoretical domains framework
UK: United Kingdom
