# Supplementary material for: What influences practitioners’ readiness to deliver psychological interventions by telephone? A qualitative study of behaviour change using the Theoretical Domains Framework
Source: BMC Psychiatry. 2020 Jul 16;20:371. doi: 10.1186/s12888-020-02761-3 (PMC7364130; doi:10.1186/s12888-020-02761-3)
Supplement: Supplementary file 1 — Additional file 1. Topic Guide. [file 12888_2020_2761_MOESM1_ESM.docx]

**Additional File 1.** Topic Guide

**Preamble**

Reminder of the purpose of study

Explanation of ethics, consent and confidentiality of interview and analysis

Structure of the interview (may be some overlap in questions and responses)

Recorder turned on

1. **How familiar are you with telephone-delivered psychological interventions?**

Prompts:

- To what extent is this part of your role?
- How often do you provide telephone-delivered psychological interventions?
- Do people you work with also do it?

1. **[Attitude] How do you feel about delivering therapy over the phone/ not talking face to face?**
2. **Do you feel as though you have the necessary skills to perform telephone-delivered psychological interventions?**

- Specific training in this area? Any training needs?

1. **Do you feel confident performing telephone-delivered psychological interventions?**

- [Coherence] How did you feel when you were first asked/if you are asked to deliver phone therapy? were your expectations valid? Have your views changed at all over time?
- Any modification to your approach for telephone-delivered interventions (compared to face to face or over time?)

1. **How does telephone therapy fit/align with the other work you do?**
2. **Do you feel supported in delivering telephone therapies in your organisation?**
3. **What are the advantages and disadvantages of performing telephone-delivered psychological interventions?**

- For you?
- For the patient?
- For the service generally?

1. **[Burden] Is it difficult/easier to deliver therapy remotely?**

- How easy is it to engage in treatment over the phone?
- What makes it easy/difficult? Why?

1. **Ethicality – what is most important to you about your therapy? What makes good therapy for you? What makes a good service? How does the phone help or hinder this?**
2. **How optimistic are you that telephone-delivered psychological interventions will be good for your patients?**

- Perceived impact on patients?
- Practitioner views on what SU think about telephone interventions?
- [Perceived effectiveness] Do you think phone therapy can work as well as face to face?

1. **Are there some patients for whom telephone-delivered interventions are:**

- More helpful?
- More difficult?

1. **Are there any specific factors that impact on the therapist-patient relationship during telephone-delivered psychological interventions?**

- e.g. tasks, bond, goals – does this differ depending on mode?
- [Self-efficacy] How easy/hard was it to get what you needed from your patients during phone therapy? How confident were you that you could work with patients over the phone?

1. **Are there any particular reasons you decide *not* to provide telephone-delivered psychological interventions?**

- If personal preference – what would convince you to use telephone interventions?

1. **To what degree does the physical working environment help or hinder your ability to perform telephone-delivered psychological interventions?**

- Do you have all the resources you need to perform telephone-delivered psychological interventions?
- To what extent are you influenced by colleagues’ decisions to perform telephone-delivered psychological interventions or not?
- Is there anything that could help you and your colleagues to provide telephone-delivered psychological interventions?

1. **Are there policies or processes in your organisation that prompt you to perform telephone-delivered psychological interventions?**

- What are the drivers behind telephone interventions? (Policy/political)
- Are there any incentives for performing telephone-delivered psychological interventions?
- How is quality assessed?
- Are there options to offer anything else? Is there a choice?
- What would encourage you to deliver telephone therapy?

1. **Are there any other factors that can potentially influence whether telephone-delivered psychological interventions are provided?**

- How do your colleagues feel about telephone therapy?

1. **How do you think patients find telephone therapy?**
2. **Does it take more or less effort to deliver telephone therapies – for a practitioner? For a service?**

- [Opportunity cost} Are there any difficulties in using the phone for therapy? Are these different to the difficulties likely to be experienced face to face? E.g., interruptions?

1. **Is there anything else that we have not covered that you would like to add?**
